# Amorphous Drug–Polymer Salts: Maximizing Proton Transfer to Enhance Stability and Release

**DOI:** 10.1021/acs.molpharmaceut.2c00942

**Published:** 2023-01-20

**Authors:** Amy Lan Neusaenger, Xin Yao, Junguang Yu, Soojin Kim, Ho-Wah Hui, Lian Huang, Chailu Que, Lian Yu

**Affiliations:** † School of Pharmacy, University of Wisconsin, Madison, Wisconsin 53705, United States; ‡ Drug Product Development, Bristol Myers Squibb, Summit, New Jersey 07901, United States; § Department of Chemistry, University of Wisconsin, Madison, Wisconsin 53706, United States

**Keywords:** amorphous drug−polymer salt, lumefantrine, poly(acrylic acid), physical stability, tropical conditions, dissolution

## Abstract

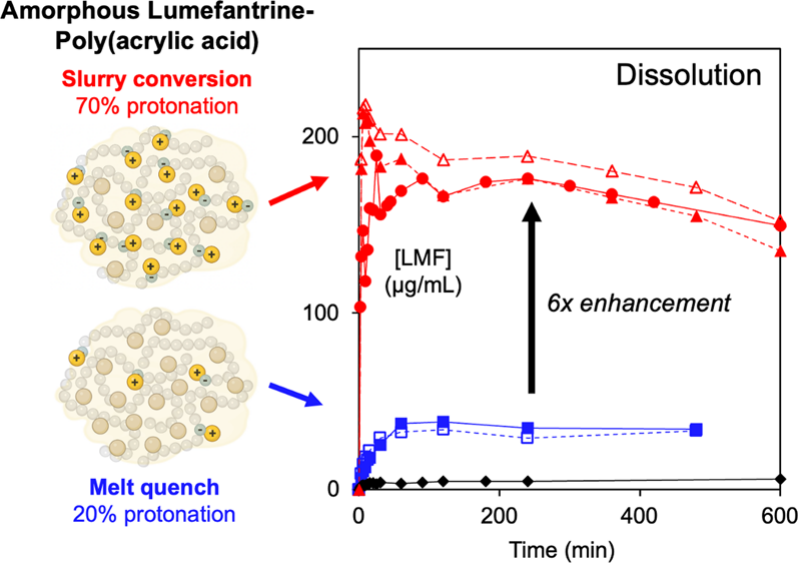

An amorphous drug–polymer salt (ADPS) can be remarkably stable against crystallization at high temperature and humidity (e.g., 40°C/75% RH) and provide fast release. Here, we report that process conditions strongly influence the degree of proton transfer (salt formation) between a drug and a polymer and in turn the product’s stability and release. For lumefantrine (LMF) formulated with poly(acrylic acid) (PAA), we first show that the amorphous materials prepared by slurry conversion and antisolvent precipitation produce a single trend in which the degree of drug protonation increases with PAA concentration from 0% for pure LMF to ∼100% above 70 wt % PAA, independent of PAA’s molecular weight (1.8, 450, and 4000 kg/mol). This profile describes the equilibrium for salt formation and can be modeled as a chemical equilibrium in which the basic molecules compete for the acidic groups on the polymer chain. Relative to this equilibrium, the literature methods of hot-melt extrusion (HME) and rotary evaporation (RE) reached much lower degrees of salt formation. For example, at 40 wt % drug loading, HME reached 5% salt formation and RE 15%, both well below the equilibrium value of 85%. This is noteworthy given the common use of HME and RE in manufacturing amorphous formulations, indicating a need for careful control of process conditions to ensure the full interaction between the drug and the polymer. This need arises due to the low mobility of macromolecules and the mutual hindrance of adjacent reaction sites. We find that a high degree of salt formation enhances drug stability and release. For example, at 50% drug loading, an HME-like formulation with 19% salt formation crystallized faster and released only 20% of the drug relative to a slurry-prepared formulation with 70% salt formation. Based on this work, we recommend slurry conversion as the method for preparing ADPS for its ability to enhance salt formation and continuously adjust drug loading. While this work focused on salt formation, the impact of process conditions on the molecular-level interactions between a drug and a polymer is likely a general issue for amorphous solid dispersions, with consequences on product stability and drug release.

## Introduction

**Scheme 1:**
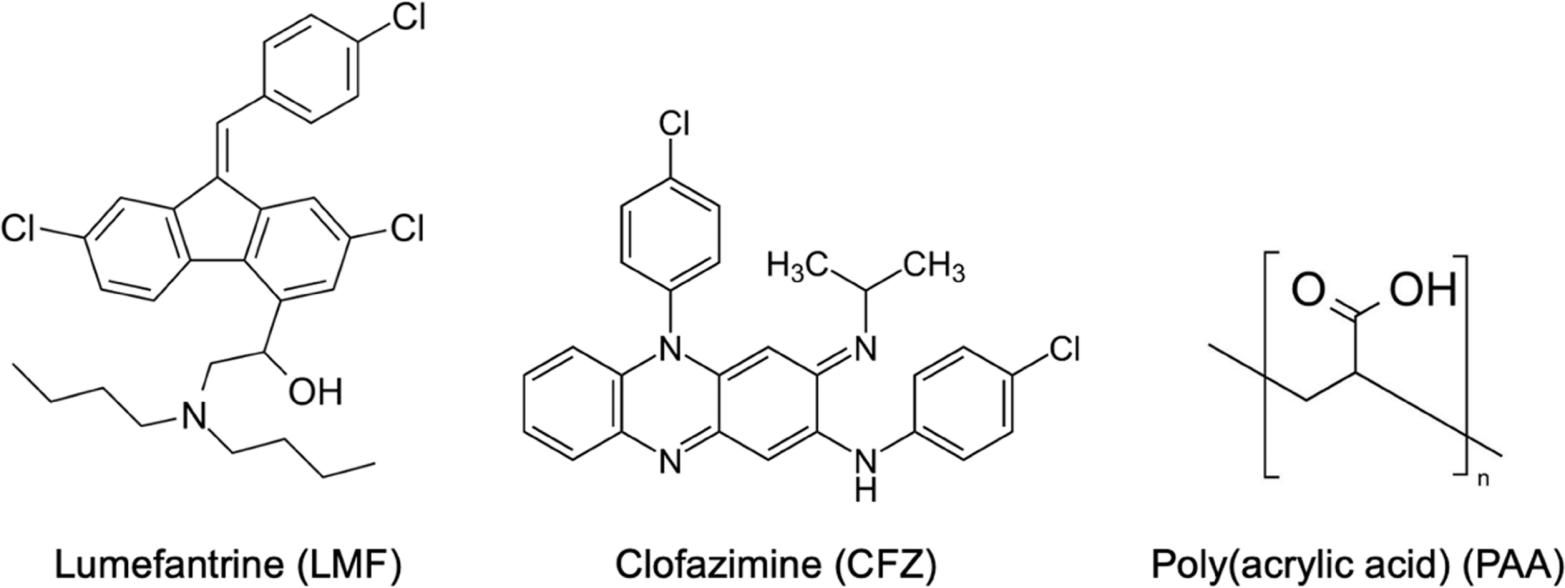
Structures of Lumefantrine (LMF), Clofazimine (CFZ), and Poly(acrylic acid) (PAA)

An amorphous solid is more soluble than its crystalline counterpart.^1,2^ In recent years, this principle has been applied to develop amorphous solid dispersions (ASDs) to deliver poorly soluble drugs.^3−5^ An ideal ASD provides enhanced solubility over its crystalline counterpart and high stability against crystallization to maintain its solubility advantage. A recent progress in this area is the formulation of amorphous drug–polymer salts (ADPS).^6,7^ An ADPS is formed by the acid–base reaction between a small-molecule drug and an oppositely charged polyelectrolyte. Relative to an ASD of neutral drug and polymer, an ADPS is more stable in a hot and humid environment, a need for many medicines for global health. This enhanced stability results from the strong ionic interaction between a drug and a polymer, which reduces the driving force for crystallization, and from the difficulty for the drug and the polymer to form a co-crystal. The increase of thermodynamic stability, at first glance, suggests reduced solubility, but excellent dissolution performance has been observed in biorelevant media for lumefantrine (LMF) and clofazimine (CFZ) formulated with poly(acrylic acid) (PAA) (see Scheme 1 for the structures of LMF, CFZ, and PAA).^6,7^

For an ADPS, the extent of acid–base reaction is a critical quality attribute. For a basic drug like LMF or CFZ, this refers to the fraction of the molecules that are protonated by an acidic polymer. Song et al. reported significant variation in the fraction of LMF molecules that were protonated by acidic polymers depending on the process condition.^8^ For example, in the formulations with PAA at 40 wt % drug loading, LMF was 5% protonated if prepared by hot-melt extrusion (HME) and 15% protonated by rotary evaporation (RE). These values indicate very low degrees of salt formation and a significant effect of the process condition. This effect is perhaps not surprising given the large size and low mobility of polymers, making a drug–polymer salt slower to form than a salt of small ions. In this work, we confirm the critical role of the process condition in forming a drug–polymer salt and demonstrate that nearly complete salt formation is possible under proper conditions.

Many methods have been used to prepare ASDs, including HME,^9,10^ spray drying^11^ (SD), and RE.^12,13^ Our recent work introduced a low-cost slurry conversion method for synthesizing ADPS.^6^ In this method, a physical mixture of the drug and the polymer is stirred in the presence of a small amount of solvent, which is then removed. Compared to SD and RE, this method uses less solvent and does not require complete dissolution of the reactants; compared to HME, it uses a lower temperature, thus applicable to thermally labile polymers such as PAA. In this work, we apply the slurry method to prepare the amorphous salt of LMF and PAA and compare the product with those prepared by HME and RE.^8^ In addition, antisolvent precipitation is tested as another method of preparation.^14,15^

Lumefantrine (LMF), the model drug of this study, is a low-solubility WHO Essential Medicine and first-line antimalarial. Jain et al. have shown that the bioavailability of LMF can be improved through an ASD formulation.^16^ Being a malaria medicine, LMF formulations should be stable under tropical conditions since many regions afflicted by malaria are hot and humid. This requirement can potentially be met using the approach of amorphous drug–polymer salts. As a weak base, LMF can be protonated by an acidic polymer like PAA.^8^ Hiew et al. investigated amorphous LMF formulated with several polymers.^17^ Their work did not include PAA and did not consider the impact of the process condition on LMF protonation, which are the focus of this study.

We report that the amorphous formulations of LMF and PAA prepared by slurry conversion and antisolvent precipitation form a single trend where the degree of drug protonation increases with PAA concentration from zero for pure LMF to ∼100% above 70 wt % PAA. This profile holds regardless of the synthetic method and the PAA molecular weight (1.8, 450, and 4000 kg/mol) and thus describes the equilibrium condition for salt formation. Remarkably, the slurry conversion method achieved much more complete salt formation than HME and RE,^8^ highlighting the importance of process conditions in completing the proton transfer between the drug and the polymer. We find that a high degree of salt formation leads to improved stability and drug release.

## Materials and Methods

### Materials

Poly(acrylic acid) (PAA, Carbomer, *M*_W_ = 1.8, 450, 4000 kg/mol) was purchased from Sigma-Aldrich (St. Louis, MO), lumefantrine (LMF) from Nanjing Bilatchem Industrial Co. (Nanjing, China), dichloromethane (ChromAR grade) from Thermo Fisher Scientific (Fair Lawn, NJ), and ethanol from Decon Laboratories (King of Prussia, PA). All materials were used as received.

### Amorphous Formulations of LMF and PAA

#### Slurry Conversion

The slurry synthesis of amorphous LMF-PAA has been described by Yao et al.^1^ In addition to the original synthesis temperature (75 °C), a reduced temperature of 25 °C was tested and we found that the products prepared after 30 min of reaction at 25 °C showed similar degrees of protonation as those prepared at 75 °C. The products were ground in an agate mortar with a pestle to a fine uniform powder prior to further analysis. For PAA of higher *M*_W_ (450 and 4000 kg/mol), a reaction with LMF was performed using both the slurry method of Yao et al.^1^ and another method with more vigorous mixing. In the latter method, a physical mixture of LMF and PAA at a chosen drug loading (25, 50, 75 wt %) was combined with the solvent (dichloromethane/ethanol, 1:1 by volume) at a 4:1 solvent/solid ratio. The resulting paste was milled in a ball mill (MM400, Retsch GmbH, Haan, Germany). The container of the mill was a 25 mL capacity steel jar with five 5 mm stainless steel balls. The mill operated at 20 Hz and the milling time was 30 min. The milling was performed at room temperature, and the internal temperature was measured immediately after milling with an IR thermometer. The increase of the internal temperature was less than 5 °C.

#### Melt Quenching

To assess the effect of the degree of salt formation on formulation performance, amorphous LMF-PAA was prepared using a melt-quench method to simulate HME. A physical mixture of LMF and PAA 450 kg/mol was prepared at 50 wt % drug and heated to 135°C while stirring with a stainless steel spatula to mimic HME. The heating time was ∼4 min. The melt was cooled to room temperature by contact with an aluminum block. The product was ground in an agate mortar with a pestle to a fine powder before further analysis.

#### Antisolvent Precipitation

A solution of LMF in acetone (50 mg/mL) was added to an aqueous solution of PAA (3.5 mg/mL) under agitation via a magnetic stir bar, causing precipitation. The precipitant was filtered using Whatman Grade 2 Qualitative Filter Paper and dried under vacuum overnight at room temperature and ground in an agate mortar with a pestle to a fine powder before further analysis.

### Powder X-ray Diffraction

X-ray diffraction patterns were collected using a Bruker D8 Advance X-ray diffractometer with a Cu Kα source operating at a tube load of 40 kV and 40 mA. A powder sample (∼10 mg) was spread and flattened on a Si (510) zero-background holder and scanned between 3 and 40° (2θ) at a step size of 0.02° and a scan rate of 1 s/step.

### X-ray Photoelectron Spectroscopy (XPS)

The details of XPS measurement and data analysis have been described previously.^18^ For an amorphous LMF-PAA formulation, approximately 5 mg of powder was pressed into a tablet using a stainless steel press. For a sample of pure LMF, approximately 1 mg of LMF powder was melted on a glass coverslip and quenched to room temperature by contact with an Al block. The samples were stored in a sealed plastic tube filled with Drierite before analysis. The high-resolution spectrum of the N atom was used to measure the fraction protonated of LMF. For each sample, the N spectrum was recorded in duplicate in two separate regions. Curve fitting was performed using the program Origin following smart baseline subtraction.

### Dissolution

Solubility tests were performed in simulated gastric fluid (SGF). The details of sample preparation, data collection, and analysis have been described previously.^6^

## Results and Discussion

### Degree of Salt Formation in Amorphous LMF-PAA Prepared by Slurry Conversion

**Figure 1:**
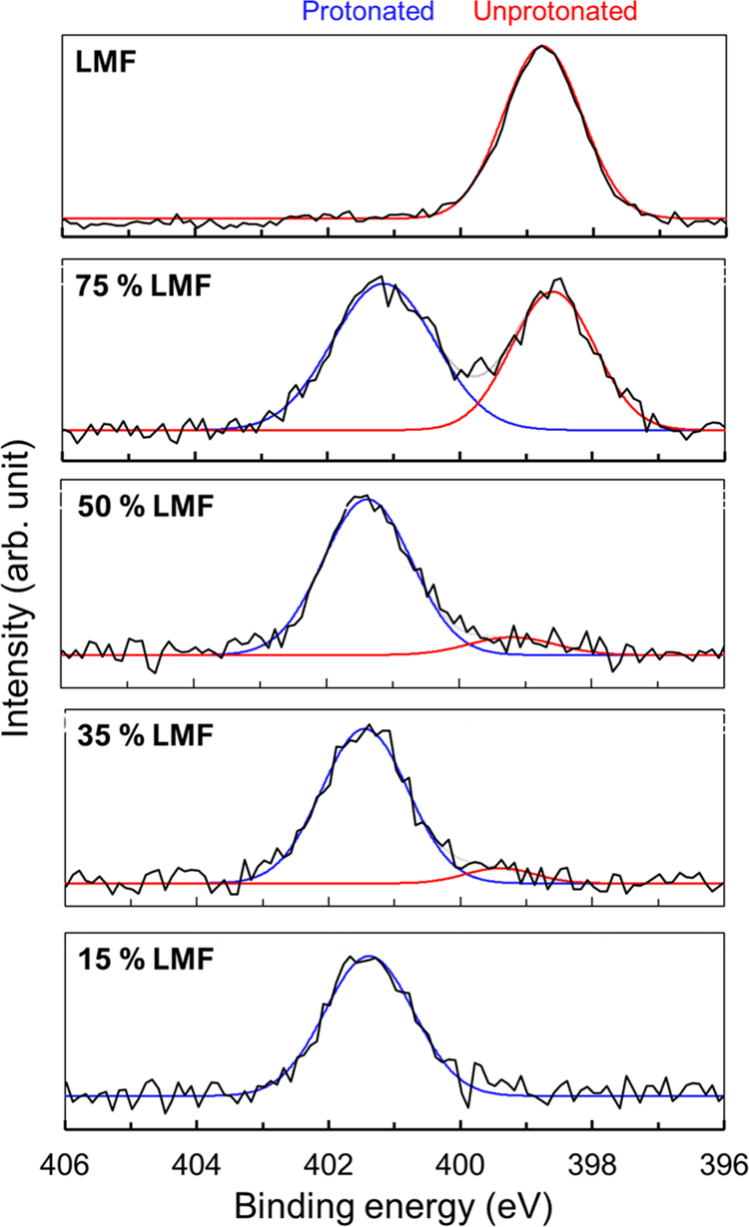
Typical XPS N spectra of amorphous LMF-PAA. These materials were prepared by slurry conversion using PAA 450 kg/mol. With increasing PAA concentration (decreasing drug loading), the unprotonated N peak decreases and the protonated N peak increases. The curves are Gaussian fits of the peaks.

Figure 1 shows the typical XPS spectra of the N atom collected to determine the degree of proton transfer (salt formation). These materials were prepared at different drug loading with PAA 450 kg/mol using the slurry conversion method and confirmed amorphous by X-ray diffraction (XRD). Yao et al. have shown^6^ that the glass transition temperatures of these materials were significantly elevated relative to those of the pure components (17 °C for LMF and 126 °C for PAA), consistent with salt formation; for example, at 50 wt % drug loading, the *T*_g_ exceeded 130 °C. The pure drug, a free base, shows a single peak at 399 eV, corresponding to the unprotonated amine N. With increasing PAA concentration (decreasing drug loading), this peak decreases and a new peak emerges at 401.5 eV. The new peak corresponds to the protonated amine group.^8,19^ Together, the spectra in Figure 1 indicate an increase in the protonated fraction of the drug with increasing PAA concentration.

The fraction protonated of LMF is calculated from an XPS spectrum as follows
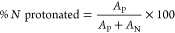
1where *A*_P_ and *A*_N_ are the areas of the protonated and the neutral N peaks, respectively, obtained by curve fitting (Figure 1).

**Figure 2:**
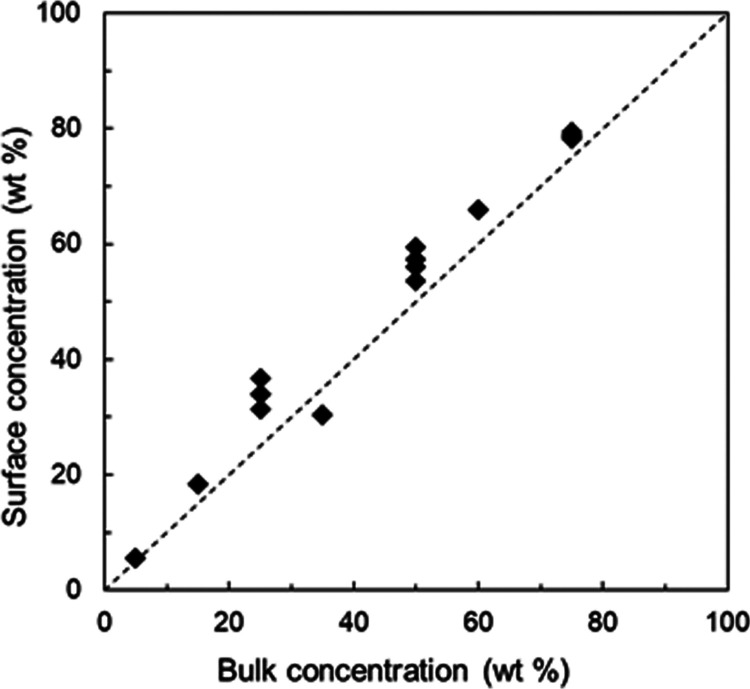
Surface concentration of LMF as a function of bulk concentration. The diagonal line indicates perfect agreement of the two concentrations.

Because XPS is a surface analytical tool with a probe depth of several nanometers,^12^ it is important to establish that the degree of salt formation measured by XPS is representative of the entire material, not just the surface region. For this, we compare in Figure 2 the drug concentrations in the bulk and at the surface for a series of materials prepared by slurry conversion. The bulk concentration was obtained from the initial amounts of LMF and PAA used for slurry synthesis. Since neither component was lost in this one-pot synthesis, the overall concentration of the product can be obtained from the initial amounts. The surface concentration was measured by XPS as follows^18^
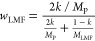
2where *w*_LMF_ is the weight fraction of LMF, *k* is the measured N/O ratio, *M*_P_ is the molecular weight of the PAA monomer, and *M*_LMF_ is the molecular weight of LMF.

Figure 2 indicates that there is no significant difference between the drug concentrations at the surface and in the bulk. This is not surprising because before XPS analysis, each sample was ground to fine particles, exposing internal surfaces. According to Yu et al.,^20^ the time for the surface composition to equilibrate is determined by the rate of polymer diffusion through the bulk and can be years or longer below the glass transition temperature. That is, even if a thermodynamic driving force exists for component segregation in the surface region, the kinetics are too slow to have a significant effect on our results and the degree of salt formation from XPS is representative of the bulk material.

**Figure 3:**
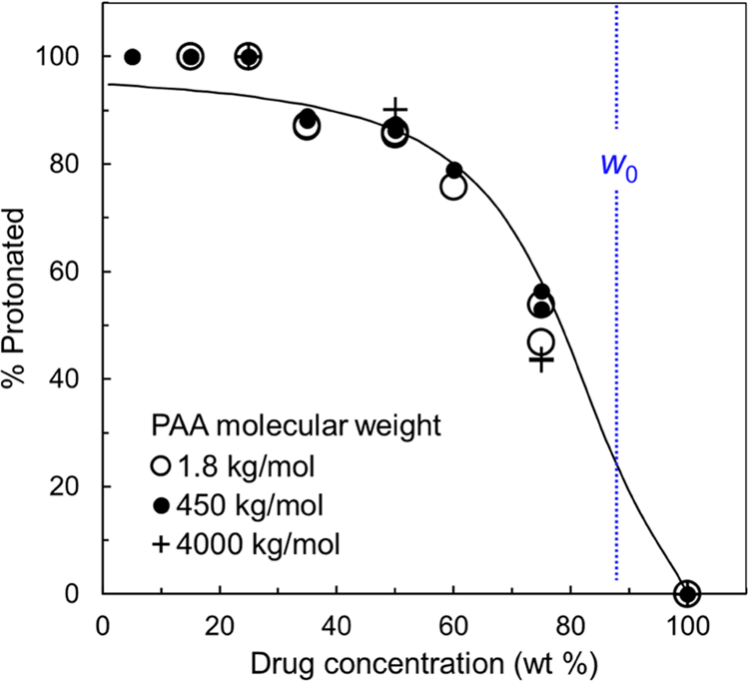
Protonated fraction of LMF in amorphous formulations with PAA of different *M*_W_s. For PAA 450 and 4000 kg/mol, some results were obtained with more vigorous mixing. A single trend is observed regardless of PAA *M*_W_, indicating the reaction had reached equilibrium. The vertical line at *w*_0_ = 88 wt % corresponds to one LMF molecule (*M*_W_ = 528.9 g/mol) per PAA monomer (*M*_W_ = 72.1 g/mol). The curve is a fit to a reaction model (see below).

Figure 3 shows the protonated fraction of LMF molecules in the amorphous formulations with PAA of three *M*_W_s (1.8, 450, and 4000 kg/mol) prepared by slurry conversion. For each *M*_W_ grade, the fraction protonated is plotted against drug loading. For PAA 1800 g/mol, the results correspond to the products of the standard slurry synthesis.^6^ For higher-*M*_W_ PAA grades, the results correspond either to the products of the standard synthesis or to those prepared with more vigorous mixing. As discussed below, for formulations of high polymer content, enhanced mixing was needed to complete the proton transfer. The data in Figure 3 form a single trend with no significant difference between PAA of different *M*_W_s. This indicates that the acid–base reaction between LMF and PAA had reached equilibrium. Had the degree of salt formation been limited by kinetics, the larger, less mobile polymer would be slower to react, resulting in less complete salt formation. The simplest explanation for the “master curve” in Figure 3 is that the slurry synthesis allowed the reaction to reach equilibrium. Consistent with this view, the curve through the data points is a fit to a reaction model (see below).

Figure 3 shows that the protonated fraction of LMF molecules increases as the PAA concentration increases (as drug loading decreases). The fraction is zero for the pure drug (a free base) and rises with the PAA concentration, approaching 100% above 70 wt % PAA. This trend is sensible since at a low PAA concentration, there are not enough acidic groups to neutralize all the basic drug molecules. The vertical line at *w*_0_ = 88 wt % corresponds to one LMF molecule (*M*_W_ = 528.9 g/mol) per PAA monomer (*M*_W_ = 72.1 g/mol). The observed profile indicates that even when PAA monomers are in excess, not every monomer can react with a drug molecule.

**Figure 4:**
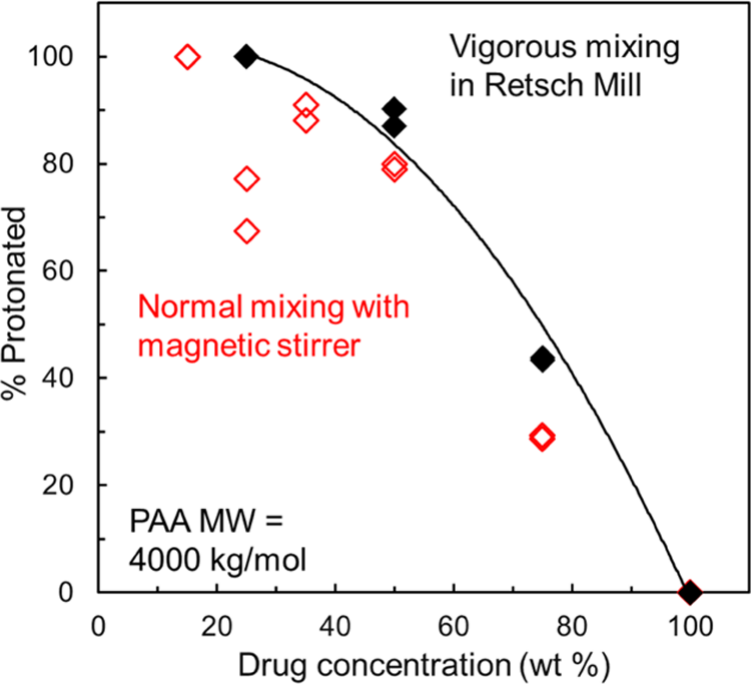
Protonated fraction of LMF in amorphous LMF-PAA prepared with PAA 4000 kg/mol using the standard slurry method (open symbols) and with enhanced mixing (solid symbols). The latter shows higher and tighter degrees of protonation due to more complete reaction. The curve is a guide to the eye.

As noted above, some formulations required more vigorous mixing to reach reaction equilibrium than utilized in the standard slurry synthesis.^6^ This occurred at higher PAA *M*_W_ and higher PAA concentration. We illustrate this in Figure 4 for PAA 4000 kg/mol. For this *M*_W_ grade, significant gelling occurred upon addition of the solvent, making stirring difficult and the reaction less reproducible. In Figure 4, we compare the protonation profiles of amorphous LMF prepared with PAA 4000 kg/mol using the standard slurry synthesis (open symbols) and with enhanced mixing in a Retsch mill (solid symbols). The standard synthesis yielded products with lower degrees of protonation and larger scatter, whereas the products formed with enhanced mixing had higher and tighter degrees of protonation. For this reason, the PAA 4000 kg/mol results in Figure 3 correspond to those obtained with enhanced mixing. A 4000 kg/mol polymer is a giant molecule, and it is not surprising that better mixing is required to complete its reaction with the drug. For PAA 450 kg/mol, the effect described above is less severe and noticeable only at high polymer concentrations (above 50 wt %). When a significant effect is noted, the results plotted in Figure 3 are those obtained with enhanced mixing.

### Amorphous Formulations of LMF and PAA by Antisolvent Precipitation

To expand the survey of synthetic methods, we investigated antisolvent precipitation as an alternative approach to preparing amorphous LMF-PAA. This method is analogous to “coprecipitated amorphous dispersion” (cPAD) of Strotman and Schenck.^9^ In this method, each component was dissolved first (LMF in acetone and PAA in water) and the mixing of the two solutions induced precipitation. The precipitant was confirmed amorphous by XRD. As in the case of slurry conversion, antisolvent precipitation was performed using PAA of different *M*_W_s (1.8, 450, 4000 kg/mol) at different drug/polymer ratios that corresponded to 25, 50, and 75% drug loading. This “bottom-up” method, in principle, enables more complete mixing of the reactants than a “top-down” method like HME and slurry conversion. An issue with the precipitation method, however, is the unknown composition of the precipitant since some reactants may remain dissolved in the supernatant. In contrast, the composition of a slurry-prepared product is known from the initial amounts of the ingredients because no ingredient is lost in the one-pot synthesis. For this reason, the drug concentration in a precipitated product must be determined and we did so by XPS from the N/O atomic ratios as described previously (Figure 2).^18^

**Figure 5:**
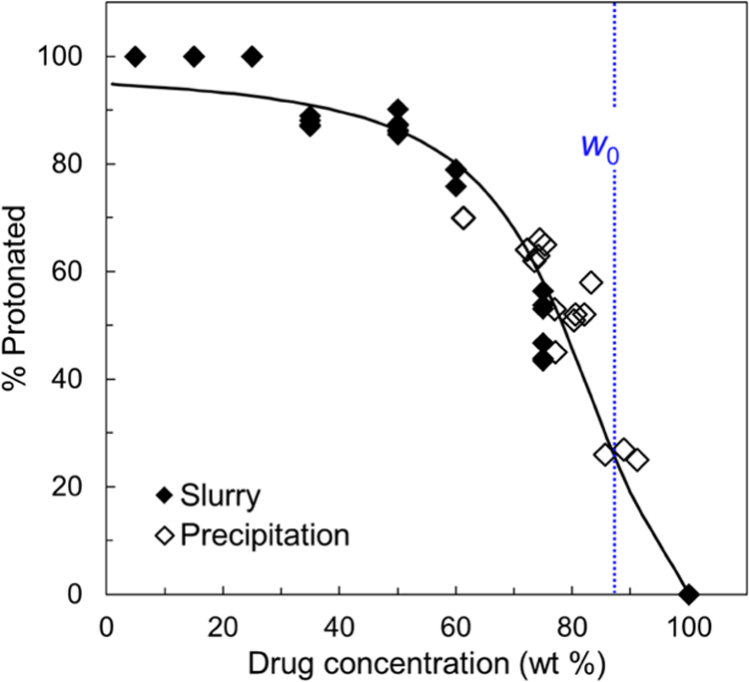
Protonated fraction of LMF vs drug concentration. The materials were prepared by slurry conversion (solid symbols) and antisolvent precipitation (open symbols) using PAA of different *M*_W_s, which are not distinguished. Within experimental error, the materials prepared by the two methods form a single trend. The vertical line at *w*_0_ has the same meaning as that in Figure 3. The curve is a fit to a reaction model (see below).

In Figure 5, we compare the protonation profiles of the products of antisolvent precipitation (open symbols) and slurry conversion (solid symbols). For the slurry products, the results are the same as those in Figure 3 but we do not distinguish the PAA *M*_W_s since the data cluster together. Similarly, for the precipitated products, the PAA *M*_W_ had no significant effect on the degree of protonation observed and we simply plot the results together without distinguishing the PAA *M*_W_s. Figure 5 shows that relative to slurry conversion, antisolvent precipitation consistently yielded products of high drug concentration (70–90 wt %), regardless of the initial drug/polymer ratio. This means a significant fraction of PAA did not precipitate with LMF but remained dissolved in the solution. This is caused by the high aqueous solubility of PAA. For this reason, the actual drug concentration in the precipitant did not correspond to the initial drug loading and must be determined post-isolation by XPS. It is interesting that the precipitated materials all had a composition close to *w*_0_ (one LMF molecule per PAA monomer).

Despite their narrower range of composition, the products of antisolvent precipitation join the same trend as those prepared by slurry conversion. This single trend supports the idea that both methods reached the equilibrium for the proton transfer between the drug and the polymer. Consistent with this view, an equilibrium reaction model yields a fitting curve that accounts for the observed data (see below). Between the two methods, slurry conversion provided continuous tunability of drug loading, whereas antisolvent precipitation yielded products of only high drug loading. For this reason, slurry conversion is the more versatile of the two and the method of choice for the remainder of this work.

**Figure 6:**
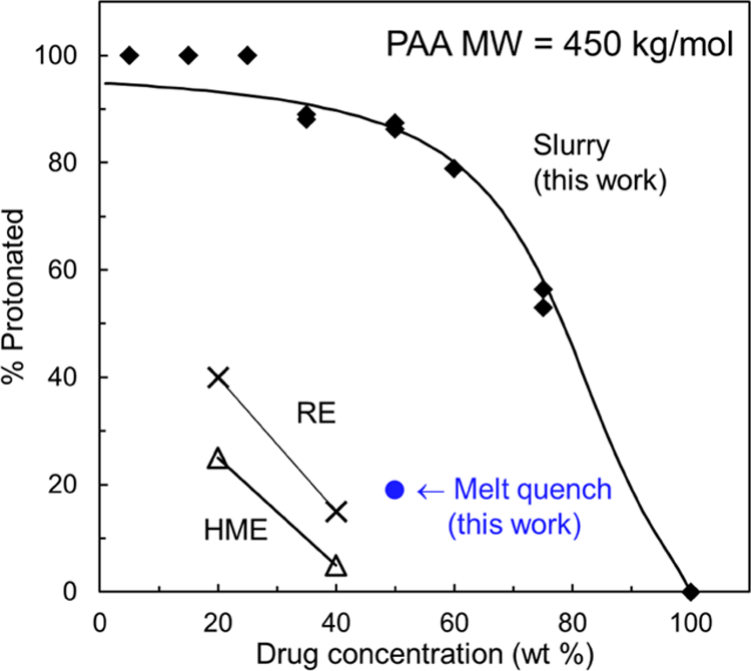
Comparison of protonation profiles in amorphous LMF formulated with PAA 450 kg/mol by different methods. At the same drug loading, slurry conversion (solid diamonds) achieved more complete salt formation than HME and RE used by Song et al.^8^ and a melt-quench method used in this work. The curve through the slurry data is a fit to a reaction model (see below).

In Figure 6, we compare the degrees of salt formation in amorphous LMF-PAA prepared by slurry conversion in this work and by HME and RE in the study of Song et al.^8^ In addition, a melt-quench formulation from this work is included. For a fair comparison, all these materials were prepared with PAA of the same *M*_W_ (450 kg/mol). All the % protonated values in Figure 6 were obtained by XPS and prior to XPS analysis, each sample was milled to ensure that the internal composition was analyzed (Figure 2). It is noteworthy that our slurry-prepared formulations reached significantly higher degrees of salt formation than those by RE and HME. At 40% drug loading, the slurry method reached 85% drug protonation, while HME and RE 5 and 15%, respectively. This indicates that the drug–polymer reaction was incomplete in the latter two cases. This result is startling since HME and RE are standard methods for ASD manufacturing and reached very low degrees of salt formation.

To investigate salt formation by HME, we prepared an amorphous formulation of LMF and PAA under conditions that mimic HME. This formulation was prepared at 50% drug loading using PAA 450 kg/mol; the ingredients were melted together and stirred in the molten state. This formulation reached 19% protonation (solid circle in Figure 6), which is broadly consistent with Song et al. HME values and significantly lower than the level reached by slurry synthesis. This comparison confirms the low degree of salt formation by HME and indicates the significant role of manufacturing methods and process conditions in completing the reaction between a drug and a polymer.

Why is the proton transfer between LMF and PAA less complete in HME than in slurry conversion? In an HME process, the components are mixed through heat and mechanical agitation without the aid of a solvent. This might suggest that a solvent could facilitate the reaction, perhaps by reducing its kinetic barrier for mass transport. This notion is consistent with Song et al. observation of a more complete salt formation by RE than by HME. However, it cannot explain the large discrepancy between their RE product and our slurry product (Figure 6). The RE process of Song et al. used more solvent (50:1 liquid/solid ratio) than our slurry method (4:1). In the RE process, LMF and PAA were initially dissolved in a single solvent (DCM/methanol), which was then removed under vacuum. The larger amount of solvent used could increase the drying time and the likelihood of phase separation during drying. Despite these differences, the similarity between RE and slurry conversion suggests that the RE conditions could be modified to achieve more complete salt formation. Overall, the results presented in Figure 6 highlight the importance of the process condition in preparing amorphous formulations that have a consistent internal state of drug–polymer interactions. Later, we will explore the effect of a varying degree of salt formation on drug stability and release.

### Model for Equilibrium Protonation Profile

**Figure 7:**
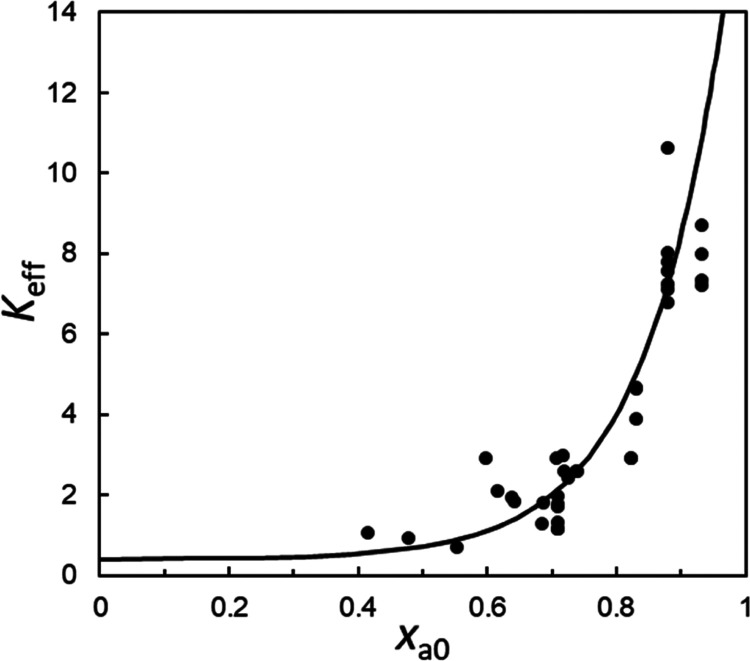
Effective equilibrium constant *K*_eff_ for the proton transfer between LMF and PAA (eq 3) vs the total AA monomer mole fraction (neutral and deprotonated) *x*_a0_. *K*_eff_ increases exponentially with *x*_a0_ (curve).

Here, we describe a model for the equilibrium protonation profile of LMF by PAA, which was used to generate the fitting curves in Figures 3, 5, and 6. Readers interested in the effect of the degree of protonation on drug performance can skip this section. This model assumes the following chemical equilibrium
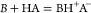
3where *B* stands for the LMF free base, HA is an average AA monomer, and BH^+^A^–^ is an ion pair between LMF and an AA monomer. The equilibrium constant of the reaction is given by
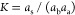
4where *a*_s_, *a*_b_, and *a*_a_ are the activities of the ion pair, the free base, and the AA monomer, respectively. Expressing concentrations as mole fractions, we have *a_i_* = *x_i_f_i_*, where *x_i_* is the mole fraction of component *i* (*i* = s, b, or a) and *f_i_* is its activity coefficient. An effective equilibrium coefficient can be defined
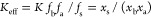
5If eq 3 represents a chemical equilibrium, *K* is a constant independent of the concentrations. But since the activity coefficients *f_i_* in general depend on concentrations, so does *K*_eff_. Figure 7 shows the experimentally determined *K*_eff_ at each drug loading from the % protonation value. We find that *K*_eff_ increases exponentially with *x*_a0_, the total AA monomer mole fraction (neutral and deprotonated). While this increase can arise from the concentration dependence of all three activity coefficients, we speculate that the coefficient for the AA monomer *f*_a_ makes the largest contribution. At a low polymer concentration, LMF molecules must compete for the reaction sites on the same polymer chain. This would be difficult, and an average AA monomer would have a low probability to react with LMF (low activity). At a high polymer concentration, many acidic groups are available to react with LMF, leading to a high probability of reaction (high activity). To generate the fitting curves in Figures 3, 5, and 6, we solve eq 5 at each drug loading with *K*_eff_ as a parameter. In addition, we assume that *K*_eff_ has an exponential dependence on *x*_a0_: *K*_eff_ = *K*_0_ + α exp(β *x*_a0_), where *K*_0_, α, and β are fitting parameters. The good fits obtained support the conclusion that slurry conversion and antisolvent precipitation can achieve the equilibrium of proton transfer between LMF and PAA.

### Effect of Salt Formation on Stability and Drug Release

**Figure 8:**
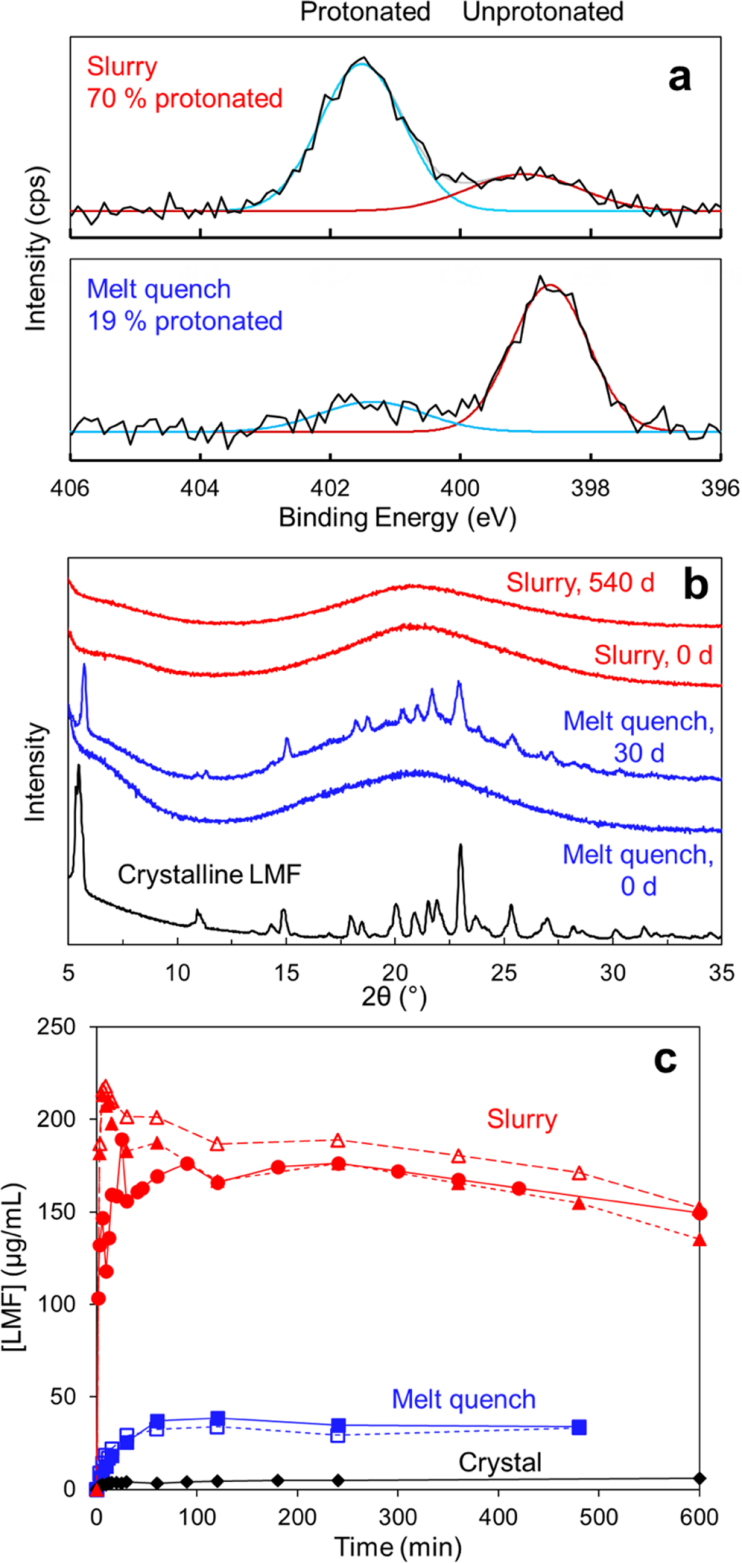
(a) XPS spectra of two amorphous LMF formulations prepared by slurry conversion and melt quench. Both were prepared with PAA *M*_W_ 450 kg/mol at 50% drug loading but had different degrees of salt formation. (b) Stability of the two formulations at (a) 40 °C and 75% RH. The melt-quenched formulation crystallized faster than the slurry-prepared formulation. (c) Dissolution curves of the two formulations in (a) and crystalline LMF. For each sample, the different symbols indicate the results on independent batches. The melt-quenched material reached higher concentration than the crystals, but much lower value than the slurry-prepared material.

To investigate the effect of salt formation on formulation performance, we studied the stability and dissolution of two amorphous LMF-PAA formulations that had identical drug loading (50 wt %) and PAA *M*_W_ (450 kg/mol), but different degrees of salt formation. By slurry synthesis, we prepared a material with 70% protonation, and by melt quench, a material with 19% protonation. Figure 8a shows the XPS spectra of these two materials. Note the prominent protonated N peak of the slurry-prepared material and the prominent unprotonated N peak for the melt-quenched material. Both materials were amorphous according to XRD.

Figure 8b shows the stability of these two materials against crystallization at 40 °C and 75% R.H. The slurry-prepared formulation remained amorphous after 540 days,^6^ whereas the melt-quenched material crystallized significantly after 30 days. This is fully consistent with our understanding of the effect of drug–polymer salt formation on stability. The salt formation between a drug and a polymer reduces the crystallization driving force to a greater extent than the mixing of a neutral drug with a neutral polymer. This comparison indicates the positive effect of more complete salt formation on stability.

Figure 8c shows the dissolution curves for the two amorphous formulations above in simulated gastric fluid (SGF). For comparison, the result is also shown for the crystalline drug. Relative to the crystals, both amorphous formulations show elevated concentrations for at least 8 h, but the slurry-prepared formulation reached significantly higher concentration (by a factor of 6) than the melt-quenched formulation. Considering their different degrees of protonation (70 and 19%, respectively), the results indicate a positive effect of salt formation on drug solubilization. It is noteworthy that the comparisons in Figure 7b,c are between two amorphous materials of identical composition, but different degrees of salt formation. This strengthens the conclusion that more complete salt formation improves the stability and the drug release of an amorphous formulation of LMF and PAA.

That the salt formation between LMF and PAA can simultaneously enhance stability and drug release might seem counterintuitive since high stability often leads to low solubility. In previous work, this dual enhancement has been observed for both LMF^6^ and CFZ^7^ formulated with PAA. Others have studied amorphous LMF formulations with polymers,^17,21^ and for a series of polymers (excluding PAA), Hiew et al. noted that RE-prepared formulations containing protonated LMF tend to be more stable against crystallization but have worse dissolution performance.^17^ Their conclusion agrees with ours with respect to stability but not dissolution. To understand this, we note that the polymer of our formulation, PAA, was not in their study and could be an outlier for their trend. In addition, the dissolution medium is SGF in this study, but a phosphate buffer in their study. Further work is warranted to develop a unified understanding.

### Greater Protonating Power of PAA “Dimer”

**Figure 9:**
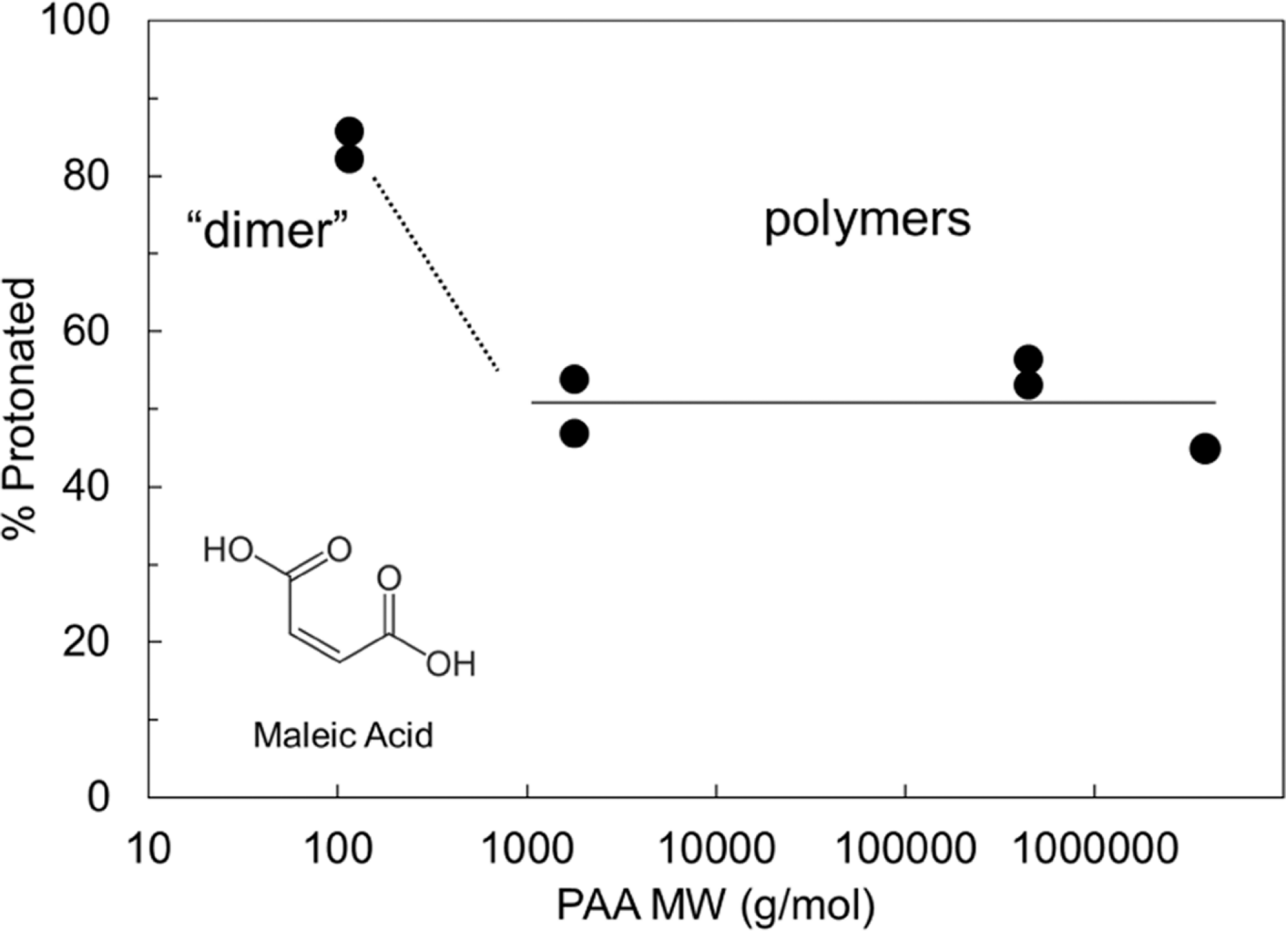
Fraction of LMF molecules in amorphous LMF-PAA that are protonated as a function of PAA *M*_W_. For this comparison, the drug loading was fixed at 75%. Maleic acid, a dicarboxylic acid, is used to mimic a PAA dimer. For the polymers, the ability to protonate LMF is insensitive to *M*_W_ (horizontal line), while the protonating power could increase for oligomers (sloping line).

The results in Figure 3 indicate that PAA of different *M*_W_s (1.8–4000 kg/mol) have similar ability to protonate LMF. We now show that at a lower *M*_W_, PAA could have a greater protonating power. Figure 9 shows the degree of salt formation as a function of PAA *M*_W_ at a fixed drug loading of 75%. At this drug loading, the polymer formulations show a similar degree of salt formation, ∼50%. We use maleic acid (*M*_W_ = 116.07 g/mol) as a mimic for a dimer of AA. An amorphous salt of LMF and maleic acid was prepared using a solvent evaporation method^22^ and was found to contain LMF that was 85% protonated. This suggests a possible increase of protonating power below *M*_W_ ∼1 kg/mol. One explanation for this effect is that LMF is a larger molecule than a PAA monomer and binding to one monomer on a polymer chain blocks access to the adjacent monomers. For a free-moving dimer, however, this crowding effect is less severe. Despite this potential increase of protonating power at low *M*_W_, we do not advocate the use of a small-molecule counterion for salt formation because we would lose the stabilizing benefit of a polyelectrolyte. Yao et al. showed that amorphous particles of LMF formulated with PAA 450 kg/mol at 50 wt % drug loading remained free-flowing after 540 days at 40 °C and 75% R.H.^6^ In contrast, the same formulation prepared with maleic acid became a viscous liquid after 1 day under the same condition. This is a consequence of a large increase in the glass transition temperature of LMF by PAA while the same stabilizing effect is not achieved with an AA dimer.

### Salt Formation in LMF-PAA and CFZ-PAA

**Figure 10:**
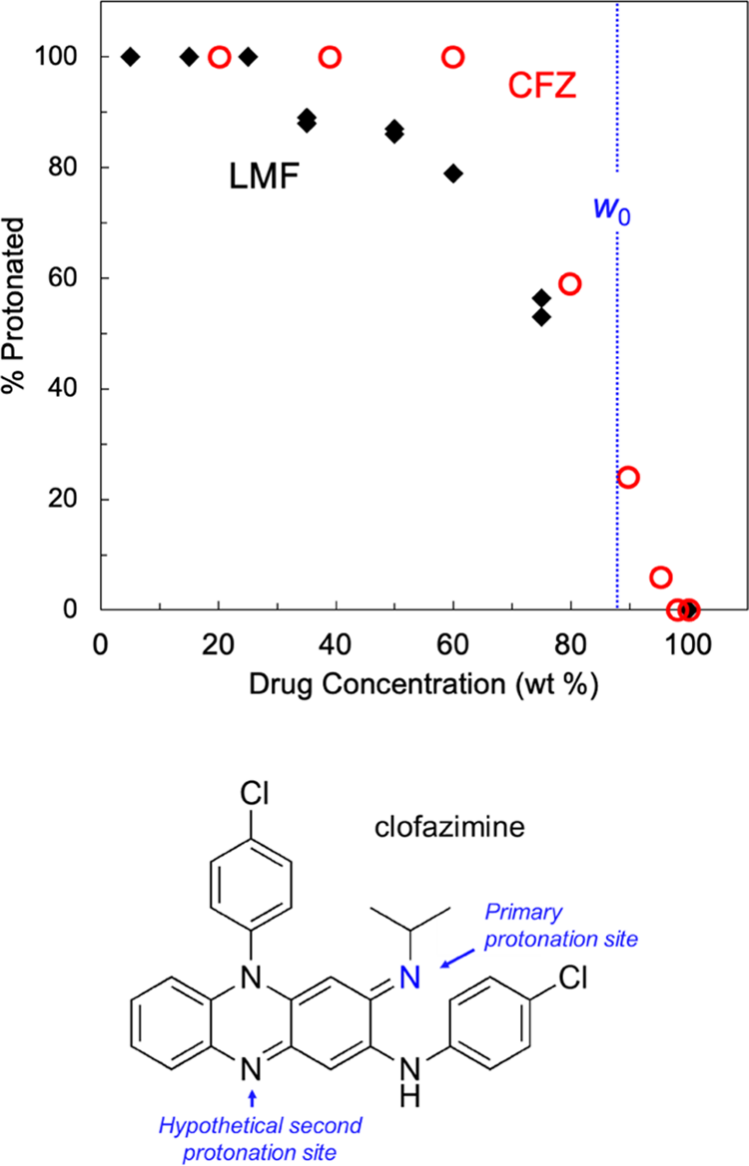
Comparison of the degrees of salt formation in LMF-PAA and in CFZ-PAA prepared with PAA 450 kg/mol. At the same drug loading, salt formation is more complete for CFZ than LMF. The vertical line at *w*_0_ corresponds to one drug molecule per PAA monomer (*M*_W_ = 72.06 g/mol) with *w*_0_ = 88 wt % for LMF (*M*_W_ = 528.9 g/mol) and 87 % for CFZ (*M*_W_ = 473.4 g/mol), indistinguishable at the scale used.

Figure 10 compares the degrees of salt formation in the LMF-PAA system and in the CFZ-PAA system.^7^ Both formulations were prepared using the slurry method with PAA 450 kg/mol. Gui et al. determined the degree of salt formation in CFZ-PAA by visible absorption spectroscopy, taking advantage of the color change of CFZ upon protonation. At the same drug loading, CFZ is protonated to a greater extent than LMF. CFZ is almost fully protonated below 60 wt % drug loading, whereas LMF does so below 30 wt % drug loading. This demonstrates the important role of the drug molecule in the degree of salt formation that can be reached. It is unclear why CFZ is more easily protonated by PAA than LMF. The literature p*K*_a_ values for the two molecules are 8.5 for LMF,^8^ and 8.4 (ref (23)) and 9.3 (ref (24), calculated value) for CFZ, which do not provide a convincing distinction of their basicity. CFZ is a marginally smaller molecule than LMF and could more easily pack around a PAA chain, perhaps facilitating salt formation. There is some evidence from spectroscopy^23^ and computer modeling^24^ that CFZ can be doubly protonated (see illustration at the bottom of Figure 10). Keswani et al. assign a p*K*_a_ of 2.3 to this site,^24^ which suggests that it could not be protonated by PAA (p*K*_a_ = 4.5). In the crystal structure of CFZ with citric acid, this site is observed to form a hydrogen bond with a carboxylic acid group without ionization, while the primary site is protonated and forms a hydrogen-bonded ion pair with a carboxylate ion.^25^ Similar multisite interactions could occur in CFZ-PAA, possibly aiding salt formation. It is interesting to note that in the crystals, the protonated LMF and CFZ each form a cyclic hydrogen-bonded ion pair with a carboxylate ion. In the fumarate salt of LMF, the ammonium group and the adjacent OH group form a cyclic hydrogen bond with both oxygen atoms of the carboxylate ion.^26^ In the carboxylate salts of CFZ, the imine N and the adjacent NH group are both hydrogen-bonded with one of the O atoms of the carboxylate ion.^26^ It is possible that similar hydrogen-bonded ion pairs occur in the amorphous phase of LMF-PAA and CFZ-PAA.

It is of interest to consider the proton transfer behavior observed in this work in light of the empirical rule for predicting salt formation from the p*K*_a_ difference, Δp*K*_a_, between the reactants.^27^ According to this rule, Δp*K*_a_ > 4 ensures salt formation. This condition is met for PAA reacting with both LMF and CFZ (primary protonation) and the rule would predict proton transfer. Experimentally, we find that proton transfer does occur in these two systems, but the degree of proton transfer depends strongly on drug loading (Figure 10). This result is not surprising given that the rule is based on a survey of small molecules. For a polymer like PAA, the reaction with one acidic group will likely hinder the reaction with adjacent acidic groups, effectively reducing their acidity and limiting the degree of proton transfer.

## Conclusions

This study investigated the effects of different synthetic methods and process conditions on the degree of salt formation between the basic drug LMF and the acidic polymer PAA. The products of slurry conversion and antisolvent precipitation form a single trend where the degree of salt formation systematically increases with increasing PAA concentration, regardless of PAA’s molecular weight (Figure 3). The master trend represents the equilibrium for salt formation since a kinetically hindered reaction would be less complete for PAA of higher molecular weight. The master trend is well described by an equilibrium reaction model (Figure 7) in further support of our conclusion. Remarkably, the literature methods of HME and RE^3^ reached far lower degrees of salt formation than the reaction equilibrium (Figure 5). This is significant since both HME and RE are standard methods for manufacturing amorphous solid dispersions. Their inability to complete the salt formation between a drug and a polymer calls for careful optimization of process conditions and characterization of the final product for quality control. We find that a high degree of salt formation has a positive effect on drug stability and release (Figure 8). Based on this work, we recommend slurry conversion as the method for preparing amorphous drug–polymer salts for its low cost, its ability to complete salt formation, and its ability to continuously adjust drug loading.

This work has provided a vivid illustration of the extremely different physical states that an amorphous drug–polymer formulation can have because of a change in manufacturing method and process condition. The amorphous nature of a formulation might give the impression that the ingredients are uniformly mixed. But for the system studied here, the drug and the polymer can be almost fully reacted to form a salt or barely reacted at all, depending on the method of preparation (Figure 6). This translates to a significant difference in drug stability and release (Figure 8). The extreme variability of physical state attained by a drug–polymer formulation stems from the low mobility of macromolecules and the linking in a chain of reaction sites. Relative to a small counterion, reaction with a polyelectrolyte could be significantly slower.^28^ Consistent with this view, in our slurry method, PAA of the highest *M*_W_ (4000 kg/mol) required more vigorous agitation to complete salt formation, especially when polymer concentration was high. Although this work focused on a system in which the drug and the polymer can ionize each other, the state of mixing is likely a general issue in developing amorphous solid dispersions, with strong impact on product performance.
